# A population-based study of 15,000 people on Knowledge and awareness of lung cancer symptoms and risk factors in Saudi Arabia

**DOI:** 10.3389/fonc.2024.1295847

**Published:** 2024-02-20

**Authors:** Saad M. AlRabeeah, Eidan M. Alzahrani, Abdulelah M. Aldhahir, Rayan A. Siraj, Abdullah A. Alqarni, Ibrahim A. AlDraiwiesh, Abdullah S. Alqahtani, Badr S. Almqati, Turki G. Alharbi, Abdulraheem A. Almuntashiri, Saeed M. Alghamdi, Fahad E. Aljohani, Mohammed A. Almulhim, Ali F. Alshehri, Abdallah Y. Naser, Hassan Alwafi, Nowaf Y. Alobaidi, Ahmed M. Hjazi, Mujahid A. Alsulaimani, Tope Oyelade, Mushabbab Alahmari, Turki M. Alanazi, Mohammed A. Almeshari, Jaber S. Alqahtani

**Affiliations:** ^1^Department of Respiratory Care, Prince Sultan Military College of Health Sciences, Dammam, Saudi Arabia; ^2^Department of Physical Therapy, Prince Sultan Military College of Health Sciences, Dammam, Saudi Arabia; ^3^Respiratory Therapy Department, Faculty of Applied Medical Sciences, Jazan University, Jazan, Saudi Arabia; ^4^Respiratory Therapy Department, King Faisal University, Al-Ahsa, Saudi Arabia; ^5^Department of Respiratory Therapy, Faculty of Medical Rehabilitation Sciences, King Abdulaziz University, Jeddah, Saudi Arabia; ^6^Respiratory Care Program, College of Applied Medical Sciences, Umm Al-Qura University, Makkah, Saudi Arabia; ^7^Pediatric Department, Khobar Governmental Hospital, Khobar, Saudi Arabia; ^8^Anesthesia Section, Security Forces Hospital Dammam, Dammam, Saudi Arabia; ^9^Preventive Medicine Department, Khobar Primary Health Care Centers, Khobar, Saudi Arabia; ^10^Department of Applied Pharmaceutical Sciences and Clinical Pharmacy, Faculty of Pharmacy, Isra University, Amman, Jordan; ^11^Faculty of Medicine, Umm Al Qura University, Mecca, Saudi Arabia; ^12^Respiratory Therapy Department, King Saud bin Abdulaziz University for Health Sciences, Alahsa, Saudi Arabia; ^13^King Abdullah International Medical Research Center, Alahsa, Saudi Arabia; ^14^Department of Medical Laboratory Sciences, Prince Sattam Bin Abdulaziz University, Al-Kharj, Saudi Arabia; ^15^Basic Medical Unit, Prince Sultan Military College of Health Sciences, Dammam, Saudi Arabia; ^16^University College London (UCL) Division of Medicine, London, United Kingdom; ^17^Department of Respiratory Therapy, University of Bisha, Bisha, Saudi Arabia; ^18^Rehabilitation Health Sciences Department, College of Applied Medical Sciences, King Saud University, Riyadh, Saudi Arabia; ^19^Institute of Inflammation and Ageing, University of Birmingham, Birmingham, United Kingdom

**Keywords:** lung cancer, Saudi Arabia, public awareness, survey, smoking

## Abstract

**Background:**

Lung cancer is currently the most fatal form of cancer worldwide, ranking as the fourth most prevalent type in Saudi Arabia, particularly among males. This trend is expected to increase with growing population, lifestyle changes, and aging population. Understanding the awareness of the Saudi population regarding the risk factors and symptoms of lung cancer is necessary to attenuate the predicted increase in cases.

**Method:**

A cross-sectional, population-based survey was performed using a previously validated questionnaire (Lung CAM). Multiple linear regression analysis was used to assess variables associated with deficiency in knowledge and awareness of risk factors and symptoms of lung cancer.

**Results:**

Majority of the 15,099 respondents were male (65%), aged between 18 and 30 years (53%), 50% of which were educated up to a bachelor’s degree level. Overall awareness of lung cancer signs and symptoms was 53%, with painful cough and coughing up blood being the best-known symptoms. Conversely, persistent shoulder pain (44%) and clubbing fingers (47%) were the least known lung cancer symptoms. Also, 60% of the respondents showed low confidence in identifying the signs and symptoms of lung cancer. The overall awareness of the risk factors for lung cancer development was 74%, with first-hand (74%) and second-hand (68%) smoking being the most known risk factors. However, only ≤ 62% know the other non-smoking risk factors. Awareness of the risk factors and symptoms of lung cancer depended on age, gender, education, marital and employment status (p < 0.001).

**Conclusion:**

Public awareness of the risk factors and symptoms of lung cancer in Saudi Arabia is inadequate and heavily dependent on education and socio-economic status. Awareness can be improved through campaigns to raise awareness about other lesser-known lung cancer risk factors and symptoms.

## Introduction

Lung cancer is the most commonly diagnosed cancer worldwide (1). In 2020, the number of new cases of lung cancer worldwide was estimated to be around 2.21 million (2). Additionally, there were approximately 1.8 million deaths attributed to this disease with around two-third of the global burden attributed to males (1–3). In Saudi Arabia, lung cancer in 2018 was ranked as the fifth most prevalent cancer among males and 12^th^ among females (4). Unfortunately, it is anticipated that the incidence rate will rise as a result of population growth, including a projected seven-fold increase in the older adult population due to increased tobacco smoking in the Saudi population (5–8). Furthermore, in Saudi Arabia, there were 504 reported lung cancer cases in 2018, accounting for 3.2% of all newly diagnosed cases among Saudis (9). Accordingly, the death rate of lung cancer in Saudi Arabia from 2009 to 2012 was 52.75% (10).

Modifiable risk factors, such as cigarette smoking, lack of physical exercise, obesity, as well as exposure to second-hand smoke, minerals, metal particles, radon, and asbestos contributes to more than half of cancer cases, including lung cancer (4, 11). Specifically, tobacco use is one of these modifiable risk factors, accounting for 80%–90% of all cases of lung cancer (12). Thus, it is crucial to have an in-depth knowledge of risk factors if the projected trend is to be attenuated. Importantly, smoking cessation has been shown by several community-based studies to substantially reduce the risk of lung cancer (13, 14). Further, the implementation of annual lung cancer screening among high-risk individuals has been strongly advised by several authoritative sources (10, 15), as it has been shown to potentially reduce the death rate by up to 20% (15).

Most patients in the Middle Eastern and Northern African regions are diagnosed with lung cancer at an advanced stage (16). Specifically, early diagnosis of lung cancer in Saudi Arabia has been achieved only in 14% of all cases (1, 17) with most cases diagnosed at a later and metastatic stage (10). This may be partly due to the lack of public awareness regarding the risk factors, early signs, and prognosis of lung cancer (18). Moreover, lung cancer can be effectively treated when diagnosed early, making awareness of its early signs extremely crucial (19). Therefore, promoting community knowledge of lung cancer, including how to identify early signs and symptoms as well as risk factors, has the potential to facilitate timely detection, mitigate the incidence of new cases, and subsequently have a favorable influence on the overall trajectory and prognosis of the illness. Therefore, the present research evaluates lung cancer awareness across the Saudi community, including knowledge of the associated risk factors and symptoms.

## Methods

### Study design and study population

This cross-sectional study aimed to evaluate the level of public knowledge and awareness of the symptoms and risk factors associated with lung cancer in Saudi Arabia. The data was collected between January 2023 and July 2023. The recruitment of participants for this study was conducted using convenience sampling method, with careful consideration of their suitability, and population distribution across the five major regions of the kingdom. Before answering the questionnaire, all participants willingly and voluntarily provided their informed consent. The purpose and goals of this study were explicitly described prior to the commencement of the survey. The inclusion criteria include being an adult individual (aged ≥ 18 years) of Saudi Arabian nationality currently residing in Saudi Arabia.

The Prince Sultan Military College of Health Sciences Institutional Review Board (IRB) approved this study (IRB-2023-RC-003). Confidential data were de-identified and removed before processing. The study followed the guidelines of the Declaration of Helsinki.

### Survey tool and distribution

We used a validated lung cancer awareness measure (17), derived from a pre-existing generic cancer awareness model. The survey instrument comprised four main sections (see **Supplementary File**). The first section collects participants’ demographic information. The second section obtains responses regarding the signs and symptoms of lung cancer. The third section asks questions about the various risk factors for lung cancer. The fourth section gauges the respondents’ level of confidence in their ability to recognize symptoms of lung cancer. The Arabic language is the official language and the predominant means of communication for most individuals residing in Saudi Arabia. Thereby, while the survey was initially designed in English language, it was subsequently forwarded to the Professional Translation Unit of Prince Sultan Military College of Health Sciences for translation into Arabic. A proficient translator specialized in the Arabic language undertook the task of retranslating the responses back into English. The translation unit used the forward–backward translation method, as recommended by the World Health Organization, to assess the two English versions (20). A subsequent pilot study was conducted on the Arabic version, in which a sample of 10 individuals from the general population was randomly recruited. The purpose of this pilot study was to evaluate the readability and suitability of the questionnaire items (21). A cut-off of ≥70% for responses was chosen in advance as an indicator of good awareness, while awareness level below 70% was deemed to be poor. The survey used a multi-channel methodology to effectively engage participants, encompassing online platforms, such as social media and WhatsApp groups, and offline distribution in public venues, such as shopping centers, cafes, and restaurants. This approach was specifically designed to ensure inclusivity by targeting individuals who could be at risk of digital exclusion.

### Power calculation

To obtain the minimum sample size, we considered a confidence interval of 98%, a margin of error of 1%, and the adult population of Saudi Arabia in 2023 of approximately 27.58 million. We also assumed a response distribution of 50%. Thus, the minimum sample size required for this study to be widely generalized was 13,523 respondents.

### Statistical analysis

To clearly represent the characteristics of the participants, a comprehensive descriptive analysis was conducted. Normality was assessed using Kolmogorov–Smirnov test. If normally distributed (parametric), data were presented as mean and SD; otherwise, median and inter-quartile range (IQR) were used. The comparisons among the various groups were evaluated using the chi-square and Fisher’s exact tests. Pearson’s correlation coefficient measure was used to examine the correlation between lung cancer risk and symptom awareness and the participants’ confidence level. Multiple linear regression analysis was used to assess variables associated with a deficiency in knowledge and awareness of lung cancer. The co-variates included in the multiple linear models were age, gender, religion, marital status, education and job status and smoking. This was done using the total score of the lung awareness measure scale for recognizing signs and symptoms and the total score of the Likert scale concerning the awareness of risk factors as dependent variables in this model. The data analysis was performed using SPSS Statistics 28 (IBM Corp., Armonk, NY), with statistical significance set to p-values less than 0.05.

## Results

### Demographic data

A total of 15,099 participants completed the survey. More than half of the responders were male (66%), aged 18–30 years old (52.5%), and had a bachelor’s degree (52%). The distribution of participants across the different provinces is as follows – eastern region: 2,702 (17.9%), western region: 3,188 (21.1%), central region: 3,200 (21.2%), northern region: 2,140 (14.2%), and southern region: 3,869 (25.6%). Nearly half of those who completed the survey were employed (46.3%). Most of the participants did not have cancer (98%), and neither did their partners (99%), close family members (96%), other family members (92%), or friends (97%). Among the respondents, 88% were non-smokers, 9% were current smokers, with a mean of 13.75 packs per year, and 3.5% had a smoking history (**Table 1**). Females accounted for 239 (18%) of the 1,317 current smokers.

**Table 1:** Demographic data and characteristics of the respondents (n = 15,099).

Variable	N (%)/mean ± SD
Gender	
Male	10076 (66%)
Female	5023 (33%)
What is your age range (year)	
18–30	7921 (52.5%)
31–40	3393 (22.5%)
41–50	2592 (17.2%)
51–60	939 (6.2%)
> 60	254 (1.68%)
Highest educational qualification	
Elementary school	179 (1.2%)
Intermediate School	278 (1.8%)
High School	4786 (31.7%)
Diploma	1592 (10.5%)
Bachelor’s	7816 (51.8%)
Master’s	354 (2.3%)
PhD	94 (0.6%)
Region	
Eastern	2702 (17.9%)
Western	3188 (21.1%)
Central	3200 (21.2%)
Northern	2140 (14.2%)
Southern	3869 (25.6%)
Current Job	
Employed	6985 (46.3%)
Unemployed	7573 (50.2%)
Retired	541 (3.6%)
Have you, your family, or friends had cancer?	
Yes	245 (2%)
No	14854 (98%)
Have you, your family, or friends had cancer? (Partner)	
Yes	162 (1%)
No	14937 (99%)
Have you, your family, or friends, had cancer? (Close family member)	
Yes	563 (4%)
No	14536 (96%)
Have you, your family, or friends, had cancer? (Other family member)	
Yes	1169 (8%)
No	13930 (92%)
Have you, your family, or friends, had cancer? (Friend)	
Yes	415 (3%)
No	14684 (97%)
Smoking status	
Ex-Smoker	536 (3.5%)
Smoker	1317 (9%)
Non-smoker	13246 (88%)
Pack per year for cigarettes	13.75 ± 15

### Knowledge, awareness, and confidence in recognizing lung cancer signs and symptoms

**Figure 1:**
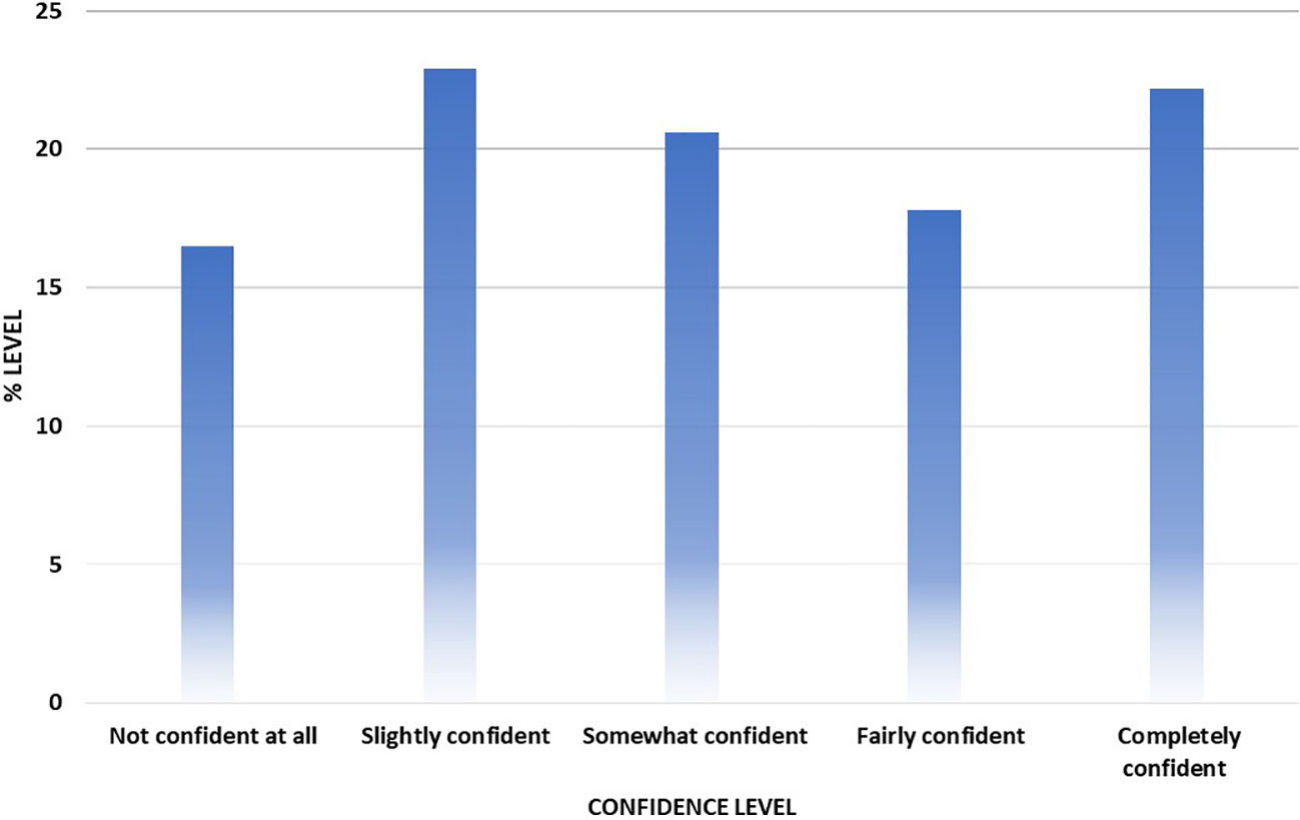
Confidence level of participants in noticing symptoms of lung cancer.

The overall level of knowledge and awareness of lung cancer signs and symptoms was 53%, which suggests poor public awareness. The least recognizable signs and symptoms were persistent shoulder pain (44%) and changes in the shape of the fingers (47%), whereas the most obvious signs were coughing up blood (65%) and painful cough (59%). Among the main lung cancer signs and symptoms, persistent cough was recognized by 55%, persistent shortness of breath by 51.5%, unexplained weight loss by 55.4%, persistent tiredness by 57%, and loss of appetite by 53% (**Table 2**). In addition, 60% of the respondents showed low confidence in identifying signs and symptoms of lung cancer (**Figure 1**).

**Table 2:** Knowledge and awareness of recognizing lung cancer signs and symptoms (n = 15,099).

Lung Cancer Awareness Measure	Yes, N (%)
1. Do you think that unexplained weight loss could be a sign of lung cancer?	8363 (55.4)
2. Do you think that a persistent (3 weeks or longer) chest infection could be a sign of lung cancer?	7999 (53)
3. Do you think that a cough that does not go away for two or three weeks could be a sign of lung cancer?	8298 (55)
4. Do you think that persistent shortness of breath could be a sign of lung cancer?	7782 (51.5)
5. Do you think that persistent tiredness or lack of energy could be a sign of lung cancer?	8627 (57.1)
6. Do you think that persistent chest pain could be a sign of lung cancer?	8346 (55.3)
7. Do you think that persistent shoulder pain could be a sign of lung cancer?	6583 (44)
8. Do you think that coughing up blood could be a sign of lung cancer?	9827 (65)
9. Do you think that an ache or pain when breathing could be a sign of lung cancer?	8302 (55)
10. Do you think that a painful cough could be a sign of lung cancer?	8952 (59)
11. Do you think that loss of appetite could be a sign of lung cancer?	8046 (53)
12. Do you think that changes in the shape of your fingers or nails could be a sign of lung cancer?	7050 (47)
13. Do you think that developing an unexplained loud, high-pitched sound when breathing could be a sign of lung cancer?	8168 (54)
14. Do you think that worsening or change in an existing cough could be a sign of lung cancer?	7276 (48)
Overall mean ± SD score out of 14	7.52 ± 3.6

### Awareness of risk factors for developing lung cancer

**Table 3** shows the awareness of the various risk factors for lung cancer among the respondents. The overall awareness level for recognizing the risk factors for lung cancer was 74%. The proportion of agreement is as follows: smoking (74%) and second-hand smoking (68%), exposure to air pollution (62%) and radon gas and chemicals (58%), having a medical history of chronic obstructive pulmonary disease (COPD) (56%), previously diagnosis with cancer (55%), having family history of lung cancer (54%), and having had treatment for any cancer in the past (49%).

**Table 3:** Awareness of the risk factors for developing lung cancer (n = 15,099).

Risk factor	Strongly Agree	Agree	Not Sure	Disagree	Strongly Disagree
1. Air pollution	5824 (38.6)	3559 (23.6)	2530 (16.8)	2235 (14.8)	951 (6.3)
2. Being a smoker	8771 (58.1)	2412 (16)	1555 (10.3)	1790 (11.9)	571 (3.8)
3. Exposure to another person’s cigarette smoke	6030 (39.9)	4237 (28.1)	1730 (11.5)	2274 (15.1)	828 (5.5)
4. Exposure to chemicals (e.g., asbestos)	5436 (36)	3292 (21.8)	3829 (25.4)	1720 (11.4)	822 (5.4)
5. Exposure to radon gas	5579 (36.9)	3160 (20.9)	4045 (26.8)	1669 (11.1)	646 (4.3)
6. Having a close relative with lung cancer	4977 (33)	3145 (20.8)	3495 (23.1)	2181 (14.4)	1301 (8.6)
7. Having a previous history of cancer, such as head and neck cancer	5723 (37.9)	2600 (17.2)	3623 (24)	2043 (13.5)	1110 (7.4)
8. Having a previous history of lung disease, such as chronic obstructive pulmonary disease	4926 (32.6)	3555 (23.5)	3539 (23.4)	1878 (12.4)	1201 (8)
9. Having had treatment for any cancer in the past	4861 (32.2)	2536 (16.8)	4213 (27.9)	2410 (16)	1079 (7.1)
Overall mean ± SD score out of 45					33.45 ± 6.24

### Determinants of lung cancer risk factors and signs/symptoms awareness

**Table 4** shows the associations between knowledge and awareness of lung cancer signs/symptoms and demographics. Being 18–30 years of age, male, living in the central and southern regions, married, employed, or having a bachelor’s degree were significantly associated with higher knowledge of the signs/symptoms of lung cancer (p < 0.001). Similarly, awareness of the risk factors of lung cancer was significantly associated with age group, gender, marital status, educational status, and smoking status (p < 0.001) (**Table 5**).

**Table 4:** Linear regression between lung cancer awareness measures and demography.

Model	B Coefficients	Std. Error	t	P-value
(Constant)	9.377	.255	36.803	<0.001
**Age group**	-.716	.028	-25.201	<0.001
**Gender**	-.537	.061	-8.746	<0.001
**Region**	-.115	.020	-5.841	<0.001
**Marital status**	-.672	.061	-11.093	<0.001
**Educational status**	.333	.027	12.146	<0.001
**Employment status**	.145	.052	2.767	<0.001
**Smoking status**	-.015	.049	-.311	0.756

Dependent variable: Lung cancer awareness measure; R = 0.252.

**Table 5:** Linear regression between risk factors awareness and demography.

Model	B Coefficients	Std. Error	t	P-value
(Constant)	35.291	.436	80.934	<0.001
**Age group**	-.899	.049	-18.484	<0.001
**Gender**	-2.260	.105	-21.500	<0.001
**Region**	-.078	.034	-2.326	.020
**Marital status**	-1.193	.104	-11.505	<0.001
**Educational status**	.781	.047	16.627	<0.001
**Employment status**	.232	.090	2.581	.010
**Smoking status**	.467	.084	5.531	<0.001

Dependent variable: Awareness of risk factors; R = 0.284.

### Correlation between lung cancer risk and symptom awareness and participants’ confidence level

**Figure 2:**
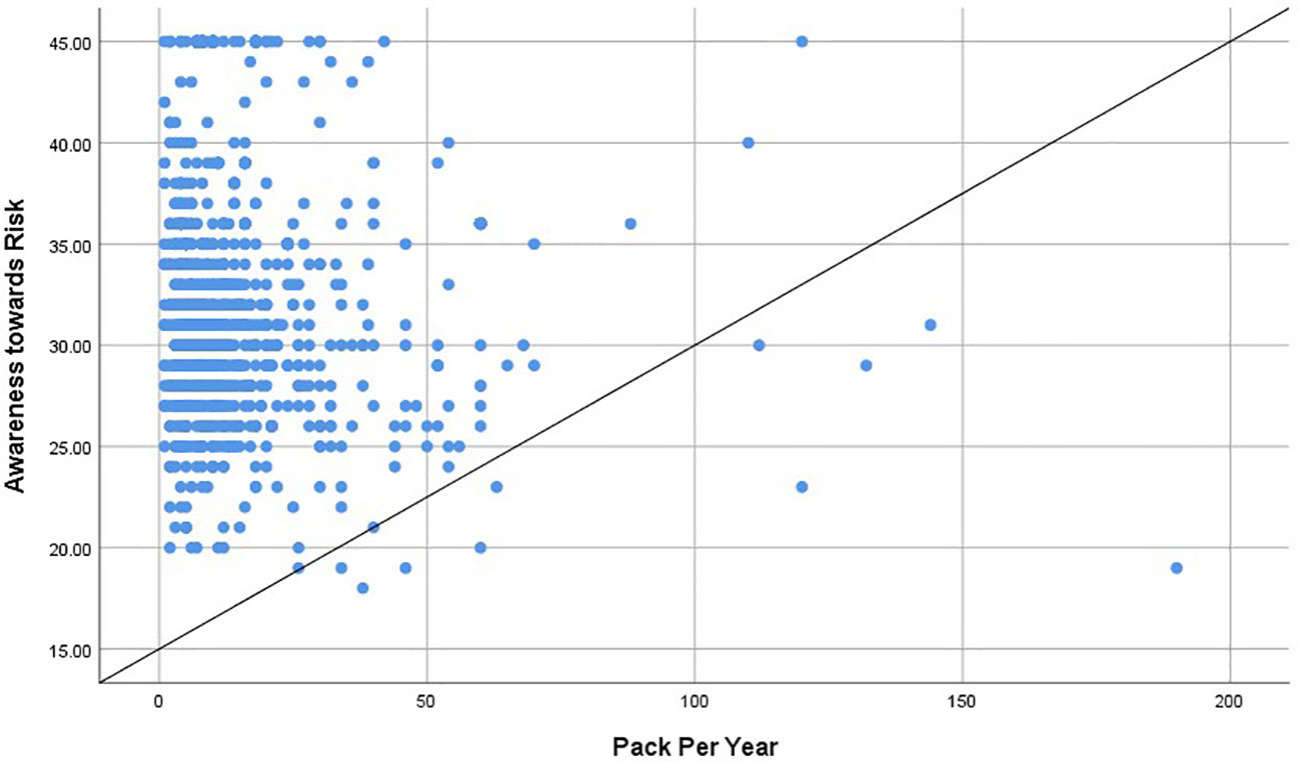
Correlation between pack per year and lung cancer awareness of risk factors (R = -.058, p-value = 0.035).

**Table 6** shows the significant positive correlation between the lung cancer awareness measure, awareness of risk factors, and level of confidence in recognizing the symptoms of lung cancer. Respondents with greater awareness of the signs and symptoms of lung cancer concurrently had better awareness of the risk factors as well as higher confidence in identifying the symptoms of lung cancer. **Figure 2** shows a statistically significant negative correlation between pack per year and lung cancer awareness of risk factors (R = −.058, p-value = 0.035).

**Table 6:** Correlation between lung cancer risk and symptom awareness and the participants’ confidence level (n = 15,099).

		Awareness of risk factors	Confidence in noticing symptoms of lung cancer
**Lung cancer awareness measure for signs and symptoms**	Pearson’s correlation coefficient	.611	.035
	Sig. (2-tailed)	<0.001	<0.001

## Discussion

In this first-of-its-kind national survey of over 15,000 participants, we assessed the public’s awareness of the risk factors and symptoms of lung cancer in the Kingdom of Saudi Arabia. Our main results show that, while more than half of the respondents recognized the main symptoms of lung cancer, such as persistent cough, shortness of breath, coughing up blood, and unexplained weight loss, they were less familiar with other symptoms such as persistent shoulder pain and finger clubbing, with around two-thirds admitting their lack of certainty (confidence) in making an association between the symptoms and lung cancer.

The finding that some of these symptoms of lung cancer are less recognized by our study population is similar to a previous report in Ireland in which morphological changes to the fingers and persistent shoulder pain were symptoms that the populace were least likely to associate with lung cancer (22). In a similar population survey performed in the United Kingdom, it was reported that only approximately 30% and 20% of the sampled population recognized persistent shoulder pain and clubbing fingers, respectively, as signs of potential lung cancer (17). This is interesting as most lung cancer patients often present to the clinic with advanced stages of the disease, usually when curative treatment is no longer applicable (23). In other studies from a neighboring country, Palestine, authors have also reported suboptimal levels of knowledge regarding symptoms of lung cancer among general population (24, 25). In a study of Saudi patients with lung cancer, the stage at diagnosis was emphasized as the single most important predictor of mortality in patients (10). This further validates how early detection through timely identification of symptoms is essential for better clinical management and prognosis of lung cancer. Indeed, the survival rate of various cancers has been shown to increase with the application of population-based screening techniques and early detection (26).

In terms of the risk factors for the development of lung cancer, most respondents showed a good level of awareness, with over 70% agreeing that smoking is a significantly increases the risk of the disease. Only 9% of the respondents in our sample were smokers, which is less than the 14% described by Qattan et al. research (27). This difference can be explained by the fact that our study had 15,099 subjects, while their study only had 8813. Indeed, more population-level education is needed to reduce or avoid the health and non-health impacts of smoking (28). Exposure to pollution, radon gas, and chemicals, as well as being diagnosed previously with COPD or other cancer types, and familial history were recognized by more than 50% of the respondents as risk factors. However, less than 50% were aware that relapse from any previously treated cancer type is a risk factor for lung cancer. This finding corroborates previous studies that found that smoking remains the most popularly known risk factor for lung cancer, with a relatively lower awareness of other risk factors (17). Our finding also supports Palestinian research that indicated smoking-related lung cancer risk factors were more generally recognized than non-smoke-related factors (25). There has also been discussions about the need to improve the awareness of non-smoking-related cancer risk factors in the general population, as most people already associate smoking with cancer. For example, Redeker et al. showed a similarly low awareness of risk factors for cancer that are not smoking-related, thereby suggesting a change in campaign focus (29). A substantial proportion of lung cancer patients has been shown to be non-smokers, and other factors, such as exposure to radon and domestic fuel smokes, have been implicated as factors that increases the risk of the disease (30, 31).

It is interesting to observe that the limited or inadequate understanding of risk factors is not exclusive to lung cancer. A recent comprehensive study conducted on a national scale examined the general population’s awareness of the risk factors associated with COPD (32). The authors revealed a generally low awareness among the studied population (32), despite high prevalence and incidence of COPD in Saudi Arabia (33). This is especially interesting as the survey was conducted in the post-COVID-19 era, with the expectation that knowledge and awareness of the risk factors for lung diseases would generally be better than before the COVID-19 pandemic.

Furthermore, we assessed the factors associated with awareness of the risk factors for lung cancer and the ability to recognize the symptoms of the disease using a linear regression model. Our results show that sufficient knowledge or awareness of the signs and symptoms of lung cancer was significantly determined by age, gender, geographical location, marital status, level of education, and employment status. Specifically, we found that being male, young (18–30 years old), living in central or southern region, being married, employed, or educated up to bachelor’s degree level increases the possibility of being aware of lung cancer signs and symptoms. Similarly, age, gender, marital status, educational level, and smoking status were found to be strong determinants of the awareness of lung cancer risk factors. These findings corroborate previous studies regarding the factors that determine awareness of lung cancer risk factors. For example, Chawla et al. found that socio-economic status and education level determined the level of awareness of the risk factors for lung cancer in a population of Nepalese residents (34). In the same study, the authors observed that while most respondents knew smoking is a risk factor, their knowledge did not influence their intention to quit smoking. This was further substantiated by another study that found that socio-economic status determined the level of awareness of the risk factors of cancer in a sample of UK residents (29). As expected, we also found that respondents with greater awareness of the risk factors for the development of lung cancer were also more aware of the predisposing symptoms and had a higher level of confidence in identifying the symptoms.

This survey has some limitations. First, there is risk of recall and self-reporting bias inherent to surveys, which may limit the interpretations of our findings (35). Second, the survey questionnaire was mainly distributed online, but we offered Offline distribution at shopping malls, cafés, and restaurants that targeted people at high risk of being digitally. Third, we lack data about household income and the results may not be applicable to a wider population due to the use of convenience sampling. Nevertheless, the study population included randomly sampled group of over 15,000 Saudi Arabians, the largest survey of its kind, to the best of our knowledge.

In sum, there is relatively low awareness of the risk factors and symptoms of lung cancer in the general population in the Kingdom of Saudi Arabia, with socio-economic status playing an influential role in this observation. The incidence and mortality due to lung and other types of cancer in Saudi Arabia are expected to increase, mostly due to social and economic development. Thus, more people are now exposed to various risk factors of cancer, including higher caloric intake, sedentary lifestyle, and smoking (28, 36). There is indeed, need to improve public awareness to change this outlook. The recent launch of Vison 2030 in the Kingdom of Saudi Arabia (37), which relates to the transformation of healthcare in the country, means that to achieve the lofty aims of reducing the national burden of diseases, more attention will need to be paid to public awareness of the various risk factors for common diseases, including lung cancer. Specifically, other significant risk factors aside from smoking should be the main target of the public health campaign, and this combined with a targeted, age-specific, sex-specific, public health screening, could help achieve a healthier Saudi Arabia. Implementing extensive public education initiatives and enhancing school curriculums to highlights the commonly known and unknown symptoms and risk factors of lung cancer may also help improve awareness.

## Conclusion

Insufficient public awareness of the risk factors and symptoms of lung cancer in Saudi Arabia was observed, with a notable dependency on educational attainment and socio-economic status as the determining factors. One potential avenue for improvement involves the implementation of targeted campaigns aimed at enhancing public awareness of the obscure risk factors for and symptoms of lung cancer.

## Data availability statement

The original contributions presented in the study are included in the article/**Supplementary Material**. Further inquiries can be directed to the corresponding author.

## Ethics statement

The studies involving humans were approved by the Prince Sultan Military College of Health Sciences Institutional Review Board (IRB-2023-RC-003). The studies were conducted in accordance with the local legislation and institutional requirements. Written informed consent to participate in this study was not required from the participants in accordance with the national legislation and the institutional requirements.

## Author contributions

SAr: Investigation, Methodology, Writing – original draft, Writing – review & editing. JA: Conceptualization, Data curation, Formal analysis, Investigation, Methodology, Project administration, Resources, Validation, Writing – original draft, Writing – review & editing. AMA: Conceptualization, Data curation, Formal analysis, Methodology, Validation, Writing – review & editing. RS: Data curation, Formal analysis, Investigation, Writing – review & editing. AAAl: Data curation, Formal analysis, Investigation, Validation, Writing – review & editing. IA: Data curation, Investigation, Validation, Visualization, Writing – review & editing. ASA: Data curation, Investigation, Visualization, Writing – review & editing. BA: Conceptualization, Data curation, Formal analysis, Resources, Writing – review & editing. TGA: Data curation, Investigation, Resources, Writing – review & editing. AAAm: Data curation, Investigation, Methodology, Project administration, Writing – review & editing. SAg: Data curation, Methodology, Validation, Visualization, Writing – review & editing. FA: Data curation, Investigation, Resources, Writing – review & editing. MAl: Data curation, Investigation, Project administration, Resources, Validation, Visualization, Writing – review & editing. AFA: Data curation, Investigation, Resources, Writing – review & editing. AN: Formal analysis, Investigation, Methodology, Validation, Visualization, Writing – review & editing. HA: Data curation, Formal analysis, Investigation, Validation, Visualization, Writing – review & editing. NA: Data curation, Investigation, Validation, Visualization, Writing – review & editing. AH: Data curation, Investigation, Project administration, Validation, Visualization, Writing – review & editing. MAs: Data curation, Investigation, Project administration, Resources, Visualization, Writing – review & editing. TO: Investigation, Methodology, Resources, Validation, Visualization, Writing – review & editing. MAa: Data curation, Investigation, Project administration, Validation, Visualization, Writing – review & editing. TMA: Data curation, Investigation, Project administration, Resources, Validation, Visualization, Writing – review & editing. MAm: Data curation, Investigation, Validation, Visualization, Writing – review & editing. EA: Data curation, Investigation, Methodology, Project administration, Resources, Validation, Visualization, Writing – review & editing.
