# Supplementary material for: A population-based study of 15,000 people on Knowledge and awareness of lung cancer symptoms and risk factors in Saudi Arabia
Source: Front Oncol. 2024 Feb 20;14:1295847. doi: 10.3389/fonc.2024.1295847 (PMC10916300; doi:10.3389/fonc.2024.1295847)
Supplement: Supplementary file 1 [file DataSheet_1.pdf]

A population-based study of 15,000 people on Knowledge and awareness of lung cancer symptoms and risk factors in Saudi Arabia

***Corresponding author:** Dr. Jaber S. Alqahtani, Department of Respiratory Care, Prince Sultan Military College of Health Sciences, Dammam 34313, Saudi Arabia; Alqahtani-Jaber@hotmail.com

Demographic Questions

First Section: -

1. What is your age?
   - 18 – 30
   - 31 – 40
   - 41 – 50
   - 51 – 60
   - >60

2. What is your gender?
   - Male
   - Female

3. Which region are you from?
   - Eastern Region
   - Western Region
   - Central Region
   - Northern Region
   - Southern Region

4. What is your marital status?
   - Single
   - Married

5. What is the highest level of education qualification you have obtained?
   - Elementary school
   - Intermediate School
   - High School
   - Diploma
   - Bachelor
   - Master
   - PhD

6. Are you currently:

- Employed
- Unemployed
- Retired

7. Have you, your family or friends had cancer?

	Yes	No
You		
Partner		
Close family member		
Other family member		
Friend		

8. Do you smoke?

- Yes, nowadays
- Yes, in the past
- No

The sub-questions related to Q8:

How many packets you smoke per a day? (If The answer is *Yes, nowadays*)

How long have you been smoking? (If The answer is *Yes, nowadays*)

How many packets you used to smoke per a day?(If The answer is *Yes, in the past*)

How long were you smoking? (If The answer is *Yes, in the past*)

Lung Cancer Awareness Measure

Second Section: -

1. The following may or may not be warning signs for lung cancer. We are interested in your opinion:

**Items**	**Yes**	**No**
1. Do you think that unexplained weight loss could be a sign of lung cancer?		
2. Do you think that a persistent (3 weeks or longer) chest infection could be a sign of lung cancer?		
3. Do you think that a cough that does not go away for two or three weeks could be a sign of lung cancer?		
4. Do you think that persistent shortness of breath could be a sign of lung cancer?		
5. Do you think that persistent tiredness or lack of energy could be a sign of lung cancer?		
6. Do you think that persistent chest pain could be a sign of lung cancer?		
7. Do you think that persistent shoulder pain could be a sign of lung cancer?		
8. Do you think that coughing up blood could be a sign of lung cancer?		
9. Do you think that an ache or pain when breathing could be a sign of lung cancer?		
10. Do you think that a painful cough could be a sign of lung cancer?		
11. Do you think that loss of appetite could be a sign of lung cancer?		
12. Do you think that changes in the shape of your fingers or nails could be a sign of lung cancer?		
13. Do you think that developing an unexplained loud, high pitched sound when breathing could be a sign of lung cancer?		
14. Do you think that worsening or change in an existing cough could be a sign of lung cancer?		

Third Section: -

3. The following may or may not increase a person's chance of developing lung cancer. How much do you agree that each of these can increase a person's chance of developing lung cancer?

	Strongly Agree	Agree	Not Sure	Disagree	Strongly Disagree
1. Exposure to radon gas (a naturally occurring radioactive gas)					
2. Exposure to another person's cigarette smoke					
3. Having had treatment for any cancer in the past					
4. Having a close relative with lung cancer					
5. Exposure to chemicals (such as asbestos)					
6. Having a previous history of cancer such as head and neck cancer					
7. Air pollution					
8. Being a smoker					
9. Having a previous history of lung disease, such as, Chronic Obstructive Pulmonary Disease (COPD)					

Fourth Section: -

How confident are you that you would notice a symptom of lung cancer?

- Not confident at all
- Slightly confident
- Somewhat confident
- Fairly confident
- Completely confident
